# Oxidative Stress in Cardiovascular Diseases

**DOI:** 10.3390/antiox9090864

**Published:** 2020-09-14

**Authors:** Emilie Dubois-Deruy, Victoriane Peugnet, Annie Turkieh, Florence Pinet

**Affiliations:** Inserm U1167, Institut Pasteur de Lille, Université de Lille, 59000 Lille, France; emilie.deruy@pasteur-lille.fr (E.D.-D.); victoriane.peugnet@pasteur-lille.fr (V.P.); ani.turkieh@pasteur-lille.fr (A.T.)

**Keywords:** reactive oxygen species, oxidative stress, antioxidant, kinase, mitochondria, cardiovascular diseases

## Abstract

Reactive oxygen species (ROS) are subcellular messengers in signal transductions pathways with both beneficial and deleterious roles. ROS are generated as a by-product of mitochondrial respiration or metabolism or by specific enzymes such as superoxide dismutases, glutathione peroxidase, catalase, peroxiredoxins, and myeloperoxidases. Under physiological conditions, the low levels of ROS production are equivalent to their detoxification, playing a major role in cellular signaling and function. In pathological situations, particularly atherosclerosis or hypertension, the release of ROS exceeds endogenous antioxidant capacity, leading to cell death. At cardiovascular levels, oxidative stress is highly implicated in myocardial infarction, ischemia/reperfusion, or heart failure. Here, we will first detail the physiological role of low ROS production in the heart and the vessels. Indeed, ROS are able to regulate multiple cardiovascular functions, such as cell proliferation, migration, and death. Second, we will investigate the implication of oxidative stress in cardiovascular diseases. Then, we will focus on ROS produced by NAPDH oxidase or during endothelial or mitochondrial dysfunction. Given the importance of oxidative stress at the cardiovascular level, antioxidant therapies could be a real benefit. In the last part of this review, we will detail the new therapeutic strategies potentially involved in cardiovascular protection and currently under study.

## 1. Introduction

Cardiovascular diseases are multifactorial disorders that represent the leading causes of death worldwide according to the World Health Organization (WHO) [1]. The physiopathology of cardiovascular diseases, mainly caused by atherosclerosis, includes remodeling of blood vessels that can result in blood flow restrictions affecting the heart and the nervous system. Cardiovascular diseases comprise several disorders such as coronary artery diseases, stroke, hypertension, heart failure, congenital heart diseases, and vascular diseases. The main risk factors for cardiovascular diseases are obesity, diabetes, cigarette smoking, a sedentary and unhealthy lifestyle, and genetic predisposition [1]. Aging is also another risk factor, since it increases cardiovascular diseases prevalence mainly due to the accumulation of oxidative damage. Indeed, oxidative stress is an important factor involved in cardiovascular diseases progression. In this review, we will first detail the physiological role of reactive oxygen species (ROS) production in the heart and the vessels. Then, we will investigate the implication of oxidative stress in cardiovascular diseases and we will focus on ROS produced by NAPDH oxidase or during endothelial or mitochondrial dysfunction. In the last part of this review, we will detail the new therapeutic strategies potentially involved in cardiovascular protection and currently under study.

## 2. General Information about Reactive Oxygen Species Production

### 2.1. Sources of ROS Production

ROS have both a beneficial as well as a deleterious role. Oxidative stress occurs whenever ROS production is higher than antioxidant capacities. ROS are generated as a byproduct of mitochondrial respiration or metabolism or by specific enzymes. Various environmental factors such as exposure to ultraviolet rays, radiation, and cigarette smoking or excessive alcohol consumption promote the production of ROS and contribute to the appearance of numerous pathologies such as cancer or cardiovascular diseases [2,3,4]. At the cardiac level, the main sources of ROS are the mitochondrial electron transport chain, the xanthine oxidase, the NADPH oxidases (NOX), and the nitric oxide (NO) synthases. Dioxygen (O_2_) is the starting point for the formation of ROS. Indeed, by capturing an electron, dioxygen causes the formation of superoxide anions (●O_2_^−^). These are the most abundant ROS in cells and are responsible for the formation of all other types of ROS, notably hydroxyl (●OH) and hydroperoxyl (●HO_2_) radicals and other non-radical species, such as hydrogen peroxide (H_2_O_2_) able to form the hydroxyl radical [5].

**Figure 1:**
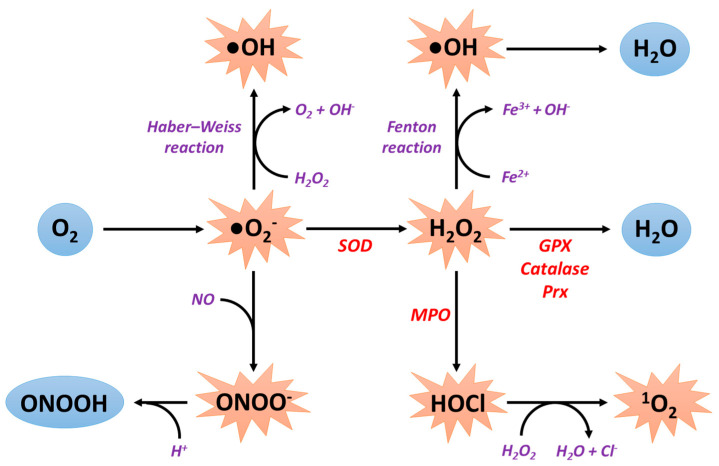
ROS formation and detoxification. ^1^O_2_: singlet oxygen; Cl^−^: chloride ion; Fe^2+^: iron (II) ion; Fe^3+^: iron (III) ion; GPX: glutathione peroxidase; H^+^: proton; H_2_O: water; H_2_O_2_: hydrogen peroxide; HOCl: hypochlorous acid; MPO: myeloperoxidases; NO: nitric oxide; O_2_: dioxygen; ●O_2_^−^: superoxide anion; ●OH: hydroxyl radical; ONOO-: peroxynitrite; ONOOH: peroxynitrous acid; Prx: peroxiredoxins; SOD: superoxide dismutases.

The superoxide anions can then interact with NO to form peroxynitrite (ONOO-) or to be converted to hydrogen peroxide by the action of superoxide dismutase (SOD) enzymes. Superoxide anions can also interact with hydrogen peroxide according to the Haber–Weiss reaction, leading to the production of the hydroxyl radical [6]. Additionally, peroxynitrite is detoxified to peroxynitrous acid (ONOOH) after capture of a proton. Hydrogen peroxide can lead to the production of hydroxyl radicals according to the Fenton reaction in the presence of ferrous ions and to the formation of hypochlorous acid (HOCl) by the action of myeloperoxidases. Hydrogen peroxide is ultimately detoxified in water by the action of several enzymes: glutathione peroxidase (GPX), catalase, and peroxiredoxins (Prx). Finally, hypochlorous acid can interact with hydrogen peroxide to form singlet oxygen (^1^O_2_) (Figure 1).

### 2.2. Antioxidant Systems

**Figure 2:**
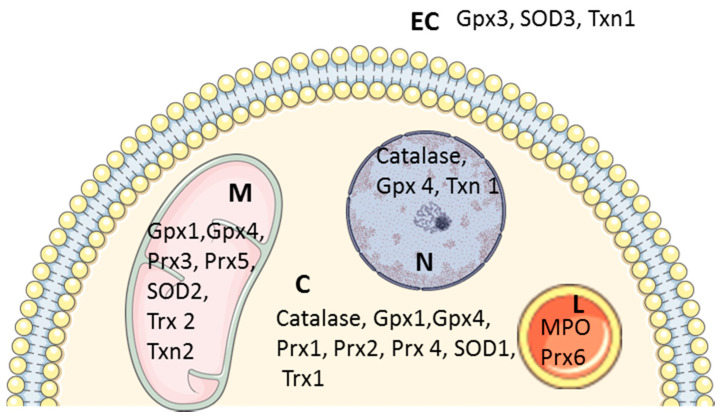
Compartmentalization of cardiac antioxidant enzymes. **C**: cytosol; **EC**: Extracellular compartment; **L**: lysosomes; **M**: mitochondria; **N**: nucleus; GPX 1–4: glutathione peroxidase 1–4; SOD 1–3: superoxide dismutase 1–3; Txn 1–2: thioredoxin 1 and 2; Trx 1–2: thioredoxin reductase 1 and 2; MPO: myeloperoxidase; Prx 1–6: peroxiredoxin 1–6.

Several antioxidant defenses protect biological systems from ROS toxicity and include antioxidant enzymes with specific compartmentalization (superoxide dismutase (SOD), catalase, glutathione peroxidase, glutathione-S-transferase, and glucose-6-phosphatedehydrogenase) and non-enzymatic antioxidants (bilirubin, α-tocopherol, and β-carotene) [7] (Figure 2).

#### 2.2.1. Superoxide Dismutases

SODs are metalloproteins able to catalyze the transformation of superoxide anion (●O_2_^−^) into hydrogen peroxide (H_2_O_2_). It is the most effective antioxidant enzyme in humans. Indeed, by detoxifying the superoxide anions, SODs inhibit their reaction with NO and prevent the formation of peroxynitrite (ONOO-). Three isoforms of SOD exist with specific subcellular localization to be closer to the source of ROS production: cytosolic SOD1, mitochondrial SOD2, and extracellular SOD3. These isoforms also require different metallic cofactors and dimerization for their activity: the SOD1 dimer and the SOD3 tetramer require a bond to copper (Cu) and to zinc (Zn), hence they are named Cu-ZnSOD, whereas the SOD2 tetramer, also called MnSOD, uses manganese (Mn) as a cofactor [8].

The implication of SOD in cardiovascular diseases has been described. As an example, inhibition of SOD2 expression induced both mitochondrial oxidative stress and cardiomyocytes hypertrophy [9]. Moreover, mice deficient in SOD2 die of cardiomyopathy within 10 days of birth, whereas heterozygous SOD2(+/−) mice show ultrastructural damage of the myocardium and mitochondria, associated to an increased oxidative stress (nitrotyrosine formation and lipid peroxidation) as well as an activation of apoptotic signaling pathways in the heart [10]. On the other hand, SOD3 activity was independently associated with abnormal left ventricular geometry patterns [11].

#### 2.2.2. Catalase

Several enzymes are able to detoxify hydrogen peroxide into water. Among them, catalase, a tetrameric heme protein, has this function depending on the concentration of hydrogen peroxide: in the case of a high concentration of hydrogen peroxide, catalytic detoxification activity is the most important, whereas in the case of a low concentration of hydrogen peroxide, peroxidase activity is the main function, with the peroxidation of various substrates such as alcohol functions or ascorbic acid [12]. Interestingly, transgenic mice expressing mitochondria-targeted catalase show an attenuation of the development of cigarette smoke or angiotensin II (AngII)-induced mitochondrial oxidative stress and hypertension compared with wild-type mice [3].

#### 2.2.3. Peroxiredoxins

Peroxiredoxins (Prx) are enzymes capable of reducing the peroxide functions of several molecules, including hydrogen peroxide and peroxynitrite. In humans, six different isoforms of Prx can be distinguished, with different subcellular locations [13] (Figure 2). At the cardiac level, peroxiredoxin 2 and 4 have been identified by proteomic analysis to be involved in cardiomyopathy and heart failure [14,15,16].

#### 2.2.4. Glutathione Peroxidases

Glutathione peroxidases (Gpx) are tetrameric selenoproteins located in the cytoplasm, the nuclei, and mitochondria and are responsible for the detoxification of hydrogen peroxide in water and the elimination of peroxide residues from lipids using the reducing capacities of the couple glutathione/glutathione disulfide (GSH/GSSG) [8]. This chain reaction system only works if GSSG is continuously reduced to GSH by glutathione reductase, itself active in the presence of NAPDH, generated by a metabolic coupling with the pentose phosphate pathway [17]. To note, Gpx4 is downregulated in the early and middle stages of myocardial infarction [18].

#### 2.2.5. Non-Enzymatic Antioxidant Defense

In general, all the proteins containing thiol groups have reducing properties and can easily trap the ROS. Glutathione is the major intracellular thiol in cells, in which it is present essentially in the reduced form. The glutathione has several antioxidant properties: it is a cofactor of Gpx, a chelator of transition metals, and the final regenerator of vitamins C and E. Glutathione can also interact with the hydroxyl radical or with the peroxide functions. Finally, the GSH/GSSG ratio is considered to be a very good marker of oxidative stress and more particularly of lipid peroxidation. Albumin is considered to be the plasma equivalent of glutathione. Furthermore, vitamin C, also called ascorbic acid, is considered to be the most effective water-soluble antioxidant in human plasma [17]. Vitamin E covers all of the tocopherol isomers, mainly represented by α-tocopherol. It is mainly imported through food, especially oils, nuts, and hazelnuts. The lipophilic nature of vitamin E as well as its location on the cell membrane gives it a powerful antioxidant action by preventing lipid peroxidation.

## 3. Physiological Roles of Oxidative Stress in Cardiovascular Tissues

**Figure 3:**
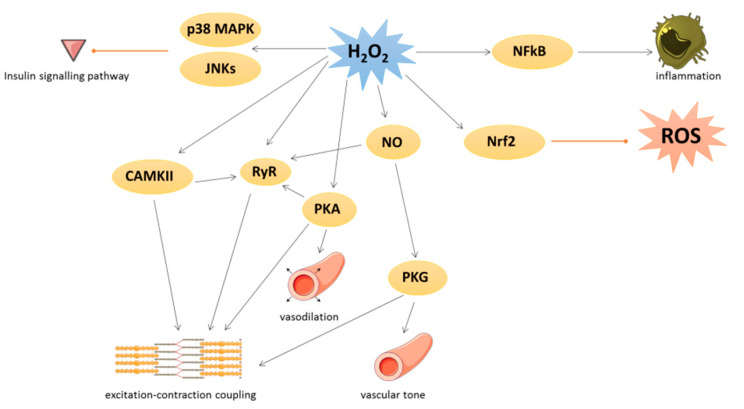
Physiological roles of oxidative stress in cardiovascular tissues. Black arrow represents activation and red arrow represents inhibition. p38 MAPK: p38 mitogen-activated protein kinase; JNK: c-Jun N-terminal kinase; H_2_O_2_: hydrogen peroxide; NFκB: nuclear factor-kappa beta; CAMKII: Ca/calmodulin-dependent kinase II; RyR: ryanodine receptor; PKA: cAMP-induced protein kinase A; NO: nitric oxide; PKG: protein kinase G; Nrf2: nuclear factor erythroid 2-related factor 2.

Under physiological conditions, low levels of ROS production are equivalent to their detoxification and play a major role in cellular signaling and function [19]. This process is called redox signaling and is defined as the specific and reversible oxidation/reduction modification of cellular signaling components able to regulate gene expression, excitation–contraction coupling, or cell growth, migration, differentiation, and death [20,21] (Figure 3).

Several kinases are involved in redox signaling. For instance, H_2_O_2_ could activate Ca/calmodulin-dependent kinase II (CAMKII), leading to excitation–contraction coupling [21] or p38 mitogen-activated protein kinase (p38 MAPK) and c-Jun N-terminal kinase (JNKs) leading to inhibition of insulin signal transduction. cAMP-induced protein kinase A (PKA) is also activated by oxidation of its regulatory subunit R1α and translocated from cytosol to membrane, where PKA regulates cardiac excitation–contraction coupling in the heart and vasodilation in the vessels [21].

Many transcription factors are also regulated by redox signaling. As an example, the nuclear factor-kappa beta (NFκB) is activated when ROS have damaged its inhibitor (IkB) and regulates inflammation process [22]. The nuclear factor erythroid 2-related factor 2 (Nrf2) could be induced by lipid peroxidation to activate several key antioxidant enzymes containing an antioxidant/electrophile response element motif in their promoter, such as heme oxygenase 1, glutathione peroxidases, SOD, peroxiredoxins, thioredoxins, and thioredoxin reductases [23]. In detail, under unstressed conditions, Nrf2 is constitutively ubiquitinated by both Kelch-k-like ECH-associated protein 1 (Keap1) and Cullin-3 E3 ligase to be damaged [23,24]. The activation of Nrf2 is due to the oxidation of Keap1 that abrogated its negative control on Nrf2.

Under physiological conditions, NO is a cytoprotective molecule with a vasodilator action. NO inhibits the activation and adhesion of platelets and neutrophils and has protective effects against ischemia reperfusion and heart failure [19,25]. NO could exert its biological effects by binding to the soluble guanylate cyclase in order to produce cyclic guanosine monophosphate leading to protein kinase G (PKG) activation [26] or by S-nitrosylation. This latter modification could modulate several protein activities such as pro-caspase 3, myosin heavy chain, tropomyosin, peroxiredoxins, or ryanodine receptor (RyR). RyR, which mediates Ca^2+^ release from sarcoplasmic reticulum, is also activated by phosphorylation via PKA and CAMKII, themselves redox-regulated [21]. Moreover, PKG is activated by oxidation independently of NO and regulates vascular tone and cardiomyocyte contraction or hypertrophy [21].

## 4. Pathological Implications of Oxidative Stress in Cardiovascular Tissues

### 4.1. General Aspects

**Figure 4:**
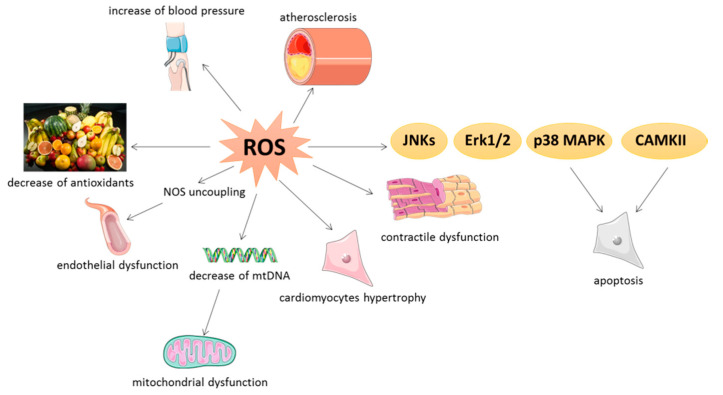
Pathological roles of oxidative stress in cardiovascular tissues. ROS: reactive oxygen species; NOS: nitric oxide synthase; mtDNA: mitochondrial DNA; JNK: c-Jun N-terminal kinase; Erk1/2: Extracellular signal-regulated kinases; p38 MAPK: p38 mitogen-activated protein kinase; CAMKII: Ca/calmodulin-dependent kinase II.

In pathological situations, ROS are able to cause oxidative modification of major cellular macromolecules (such as lipids, proteins, or DNA). This oxidation induces modifications in subcellular organelles such as sarcolemma, mitochondria, sarcoplasmic reticulum, and nucleus. As an example, ROS could modulate contractility by oxidation of sarco/endoplasmic reticulum Ca^2+^-ATPase (SERCA 2A) [27] and contractile proteins such as tropomyosin and actin, leading to contractile dysfunction [28] (Figure 4).

The toxicity of radical species depends on their half-life (the shorter they are, the more ROS are unstable and toxic) as well as their site of production (less diffusion of the ROS induces more oxidation of the non-specifically surrounding molecules). The toxicity of non-radical ROS depends on their capacity to generate radical species. Thus, the hydroxyl radical constitutes the most unstable radical ROS but with the strongest toxicity [5].

An increase in ROS production has already been described in several cardiac diseases such as myocardial fibrosis [29], type 2 diabetes [30,31], metabolic syndrome [32,33,34,35,36], cardiac hypertrophy [9,37,38], heart failure [39], and myocardial infarction [40,41].

### 4.2. NOX-Dependent Effects

NADPH oxidases (NOX) constitute a family of membrane enzymatic complexes whose primary function is the production of ROS. NOX catalyze the reduction in dioxygen to superoxide anion using NADPH or NADH as an electron donor. Seven NOX family members are initially described in neutrophils (NOX 1–5 and dual oxidase (DUOX) 1–2) with specific catalytic subunits and partners. NOX3 isoform is exclusively expressed in the inner ear, while the DUOX1 and 2 isoforms are particularly found in the thyroid, the pulmonary epithelium, and the colon. Only NOX1, 2, 4, and 5 are particularly expressed in the cardiovascular system. In detail, human aortic smooth muscle cells express NOX1, 4, and 5. NOX2 and 4 are also expressed in several cardiac cell types, such as cardiomyocytes, fibroblasts, endothelial cells, or smooth muscle cells. NOX2 is activated during cardiovascular stress induced by AngII, endothelin-1, growth factors (e.g., platelet-derived growth factor (PDGF)), cytokines, or mechanical forces, whereas NOX2 is constitutively active but increases with hypoxia, ischemia, or pressure overload [21]. All the vascular NOX (e.g., NOX1, NOX2, NOX4, and NOX5) are regulated by AngII that increases the blood pressure and NOX-dependent ROS, which in turn, activate the AngII receptor type 1 (AT-1) with an auto amplificatory effect [42]. Furthermore, AngII have pro-hypertrophic and pro-fibrotic effect in cardiac cells, notably by endothelin-1 release [43] (Figure 4).

Moreover, the level of superoxide anions is highly increased in a rat model of type 2 diabetes and associated with a decrease in both global SOD and glutathione peroxidase activities and endothelial, but not cardiac, dysfunction which could be explained by the NOX, constituting the main source of superoxide anion in vessels [30]. On the other hand, NOX activity is increased in patients with a metabolic syndrome as well as plasma levels of oxidized low density lipoprotein and nitrotyrosine [32] and a correlation between NOX mRNA expression and the severity of atherosclerotic lesions has been shown in human coronary arteries [44]. At the cardiac level, NOX-dependent superoxide anion production is increased in left ventricle of guinea pig after 3 weeks of aorta binding, leading to the activation of extracellular signal-regulated kinase 1/2 (Erk1/2), JNKs, and p38 MAPK [37]. Excessive NOX2-derived ROS has been described to activate apoptosis by ASK-1/p38MAPK and the CAMKII pro-apoptotic pathway after myocardial infarction or AngII stimulation [45] (Figure 4).

### 4.3. Endothelial Dysfunction

Endothelial dysfunction, including hypertension and atherosclerosis, is the main risk factor for stroke, myocardial infarction, and heart failure. Endothelial dysfunction is defined as a decreased production and availability of NO, with or without an imbalance between endothelium-derived contracting and relaxing factors associated with a pro-inflammatory and prothrombotic status [46]. Under oxidative stress, ROS are able to mediate endothelial dysfunction and vascular abnormalities by disrupting the vasoprotective NO signaling pathway, leading to NO synthase (NOS) uncoupling. NOS are the enzymes responsible for the synthesis of NO from L-arginine in the presence of cofactors and dioxygen, but they are also able to produce superoxide anions. Three NOS have been identified: type I or neuronal NOS (NOS1 or nNOS), type II or inducible NOS (NOS2 or iNOS), and type III or endothelial NOS (NOS3 or eNOS). NOS1 and NOS3 isoforms are constitutively expressed in the heart, more particularly in striated muscle and endothelial cells, and possess calcium-dependent activity. The NOS2 isoform has calcium-independent activity. It is not constitutively expressed in the healthy heart but it is expressed in pathological conditions such as inflammation [25,47]. When NOS are uncoupled, they switch from NO to superoxide anion production (●O_2_^−^) and peroxynitrite (ONOO-) by the association of the two preceding, leading to reduced bioavailability of NO and vasoconstriction [42] (Figure 4). Peroxynitrite is involved in atherosclerosis progression by inhibition of vasorelaxation, decrease in the beneficial effects of NO on platelet aggregation and vascular smooth muscle cell proliferation, and oxidation of DNA and lipids [48]. Moreover, aging increases oxidative stress in resistance arteries by increasing peroxynitrite production in the serum of aged mice [49]. On the other hand, mice exposed to cigarette smoking and low doses of AngII show severe endothelial dysfunction, particularly regarding acetylcholine-dependent relaxation, associated with an inactivation of SOD2 activity [3]. Moreover, heart failure also induces an increase in superoxide anions produced by NOS and NOX in vessels from cardiomyopathic hamsters [50] (Figure 4).

### 4.4. Mitochondrial Oxidative Stress

Although different sources contribute to global oxidative stress, the vast majority of cellular ROS (90%) came from the mitochondrial compartment. Indeed, during oxygen consumption, the transfer of electrons in the electron transfer chain is usually not completed and a small minority (<1%) of electrons can cause the reduction in dioxygen (O_2_) into superoxide anion (●O_2_). The use of inhibitors of the mitochondrial respiratory chain such as rotenone and antimycin, as well as the substrates for the various complexes such as pyruvate or succinate, have let to the identification of the different complexes involved. To date, complexes I, II, and III have been identified as production sites for superoxide anions [51,52].

Excessive ROS production occurs during mitochondrial dysfunction and induces irreversible damage to mitochondria, becoming significant contributors to the development of cardiovascular disease [53]. For example, an increase in mitochondrial ROS production has been described in a murine model of myocardial infarction induced by 4 weeks of coronary ligation [40]. In this model, hydroxyl radicals (●OH) from mitochondria are increased, inducing a significant decrease in mitochondrial DNA (mtDNA) as well as a significant decrease in mtDNA-encoded gene transcripts for respiratory complexes I, III, and IV [40]. Indeed, mitochondria are also a main target of ROS, in particular mtDNA, which is highly sensitive to oxidative damages notably due to its low repair capacity and proximity with the electron transfer chain [54]. On the other hand, ROS generated by AngII stimulation induce mitochondrial dysfunction, mtDNA lesions, cardiomyocytes hypertrophy, cardiac fibrosis, and heart failure [38]. Mitochondrial ROS also oxidize proteins from complex I and II of the electron transfer chain, leading to a decrease in mitochondrial respiration. For example, dysfunctions of the various complexes of the mitochondrial respiratory chain are also observed in the non-infarcted zone of the myocardium in in vivo models of myocardial infarction, associated with a decrease in mitochondrial respiration [55]. These disruptions to the mitochondrial respiratory chain are associated with increased oxidative stress and ROS production, leading to the activation of many protein kinases and transcription factors involved in hypertrophic signaling [56] (Figure 4).

Mitochondrial ROS production is also involved in many diabetes-related cardiovascular complications. Indeed, metabolic syndrome, characterized by diabetes and obesity, is associated with left ventricular hypertrophy and metabolic and diastolic dysfunction [34,36]. For example, mice fed with a high-fat high-sucrose diet develop mitochondrial oxidative stress (H_2_O_2_ production), mitochondrial dysfunction (decreased ATP synthesis, complex II activity and mitochondrial respiration), and cardiac hypertrophy (increased wall thickness) [33,34]. Moreover, an increase in mitochondrial H_2_O_2_ production is also observed in atria from diabetic patients [31]. The level of oxidative stress as well as apoptosis is also increased in right atrial cardiomyocytes obtained from obese patients [35]. On the other hand, an increase in mitochondrial superoxide anion production during cardiomyocytes hypertrophy induced by isoproterenol [9] or AngII [38] has already been described.

## 5. New Therapeutic Strategies

Since it has been established that oxidative stress plays an important role in cardiovascular pathologies, several studies have been conducted to investigate the therapeutic effects of antioxidant therapy. If many studies showed a protective effect in cellular or animal models, large clinical trials failed until now to demonstrate any benefits of antioxidant therapeutic strategies.

### 5.1. Vitamins

The therapeutic potential of several vitamins has been tested and some benefits have been observed in experimental models. Indeed, vitamins, notably vitamin C or folic acid, could prevent NOS uncoupling and inhibit endothelial dysfunction, leading to cardioprotection [57,58]. On the other hand, it was described that very high blood levels of total homocysteine, an amino acid intermediate in methionine metabolism, induced atherosclerosis and thromboembolic events, a phenomenon called “homocysteine hypothesis” [59]. Indeed, hyperhomocysteinemia causes oxidative stress and endothelial dysfunction [58,60]. Total homocysteine is significantly reduced by supplementation with folic acid, vitamin B12, and B6. For instance, folic acid improves endothelial function in vitro by reducing oxidative stress [61] and folic acid and vitamin B (12) supplementation on isoprenaline; induced myocardial infarction in hyperhomocysteinemic rats decreases oxidative stress and improve cardiac function, such as heart rate, ST segment elevation, and blood pressure [62]. Vitamin D also improved cardiac oxidative stress and inflammatory markers in obese rats [63] and a combination of vitamin E and apelin decreased apoptosis, oxidative stress, hypertrophy, and fibrosis in a mouse model of isoproterenol-induced cardiopathy, whereas vitamin E alone failed to affect cardiac remodeling [64]. On the contrary, α-tocopherol, the strongest antioxidant form of vitamin E, significantly reduced ROS (notably, the lipid peroxidation) but also the infarct size and restored cardiac function (such as ejection fraction, fractional shortening, cardiac output, and stroke volume) [65].

### 5.2. Polyphenols

Polyphenols are secondary metabolites of plants, characterized by the presence of large multiples of phenol structural units, founded in fruits (such as grapes, apple, pear, cherries, and berries), vegetables, cereals, tea, and coffee. An important number of mechanistic studies have demonstrated that polyphenols possess antioxidant or anti-inflammatory properties associated with cardioprotection and are detailed in [66]. For example, dietary supplementation of red wine polyphenol extracts reduced cholesterol and improved glucose metabolism, cardiac performance, and endothelial dysfunction in Zucker Fatty (ZF) rats [67]. Polyphenols, such as resveratrol, a derivative of stilbene, also induce the expression of antioxidant enzymes and their substrates, thereby contributing to the overall reduction in oxidative stress [68]. Resveratrol has been described to have several cardioprotective effects, detailed in [66,68,69]. Notably, it could prevent endothelial dysfunction and inflammation and increase the expression and activation of eNOS, contributing to the prevention of hypertension in obese mice [70]. Resveratrol could also reduce phenylephrine-induced protein synthesis and cell hypertrophy in rat cardiac myocytes [71]. In mice subjected to transverse aortic constriction to induce heart failure, resveratrol significantly increased survival and decreased cardiac fibrosis and markers for hypertrophy and extracellular matrix remodeling and improved diastolic function [72]. Lastly, the oral administration of resveratrol to hamsters increased SOD2 activation by increasing sirtuin 1 expression in cardiomyocytes, suppressed fibrosis, preserved cardiac function, and significantly improved survival [73]. Altogether, these data suggest that polyphenols could be novel therapeutic tools for the treatment of cardiovascular disease.

### 5.3. Mitochondrial-Targeted Antioxidant

An interesting and promising strategy could be to target mitochondria, the most important compartment involved in ROS production, and several strategies targeting mitochondria, such as small molecules and mitochondria-targeting peptides and antioxidants have been tested in preclinical and clinical studies in cardiovascular diseases [74,75]. To this end, mitoTEMPO, a mitochondria-targeted superoxide dismutase mimetic, was developed in 2010 [76]. In Vitro, mitoTEMPO decreases the mitochondrial superoxide anion and H_2_O_2_ production induced by AngII in human aortic endothelial cells [76] or adult mice cardiomyocytes treated with high glucose [77]. Several studies analyze the in vivo effect of mitoTEMPO. For example, treatment of mice with mitoTEMPO reduces hypertension induced by both AngII or deoxycorticosterone acetate (DOCA) salt [76], reduces the mitochondrial superoxide anion and 3-nitrotyrosine, serum glucose levels and diastolic dysfunction observed in high-fat diet mice [34], decreases mitochondrial ROS production, and prevents cardiomyocytes hypertrophy in diabetic mouse hearts [77]. On the other hand, mitoquinone, which comprises an exogenous ubiquinone linked to a triphenylphosphonium lipophilic cation, was developed in 2001 [78] and has been demonstrated to effectively improve mitochondrial function and attenuate redox-related cardiomyopathies [36,79]. Moreover, some studies, notably in cancer cells, described that mitoquinone could also lead to ROS production, rapid membrane depolarization, and apoptotic cell death [80,81,82].

### 5.4. Clinical Trials

Due to the preclinical effects of antioxidants, many randomized clinical trials have been performed over the past decade in both healthy and diseased humans. Unfortunately, most of these clinical studies failed to show an improvement of vitamins supplementation at the cardiovascular level [57,83,84] or even reported an increased incidence of chronic congestive heart failure in humans [85,86]. These negative effects could be explained notably by the absence of site-specificity of these molecules regarding the source of ROS production (mitochondria, for example). Another explanation could be the reactivity: for example, the reaction between vitamin C and superoxide anion is 10,000 lower than between superoxide anion and NO (leading to peroxynitrite (ONOO-)), requiring an important dose to be efficient not compatible with oral administration [57,87].

Moreover, the human clinical studies available so far have shown controversial results concerning the protective effects of resveratrol against cardiovascular diseases and are extensively detailed in [69,88]. Briefly, resveratrol could decrease inflammation [89,90,91] and endothelial dysfunction [92,93] in several studies, whereas some others have shown no effect [94,95,96], probably due to the fact that many of these trials use highly variable protocols and doses of resveratrol and suffer from small sample sizes.

Finally, numerous randomized studies are currently in progress to evaluate the effects of dietary supplementation with mitoquinone in several conditions, including diastolic dysfunction (NCT03586414), peripheral arterial disease (NCT03506633), or chronic kidney disease (NCT02364648) [46], but the impact of mitoquinone supplementation in patients remains to be determined.

## 6. Conclusions

Increased oxidative stress is one of the potential common etiologies in various cardiovascular diseases. These diseases are very complex in their pathogenesis and no single mechanism explains their physiopathology. Thus, not surprisingly, many clinical trials investigating antioxidants have been negative, whereas improving mitochondrial functionality by using mitochondria-targeted therapeutics might be an important strategy to enhance the effectiveness of non-pharmacological therapies, such as exercise. Exercise is a well-known and very effective intervention, improving cardiac mitochondrial metabolism in both health and diseases. These results should encourage scientists to continue their research in the field of oxidative stress and antioxidants.

## References

[1] Benjamin E.J., Blaha M.J., Chiuve S.E., Cushman M., Das S.R., Deo R., de Ferranti S.D., Floyd J., Fornage M., Gillespie C. (2017). Heart Disease and Stroke Statistics-2017 Update: A Report From the American Heart Association. Circulation.

[2] Al Hariri M., Zibara K., Farhat W., Hashem Y., Soudani N., Al Ibrahim F., Hamade E., Zeidan A., Husari A., Kobeissy F. (2016). Cigarette sImoking-induced cardiac hypertrophy, vascular inflammation and injury are attenuated by antioxidant supplementation in an animal model. Front. Pharmacol..

[3] Dikalov S., Itani H., Richmond B., Vergeade A., Rahman S.M.J., Boutaud O., Blackwell T., Massion P.P., Harrison D.G., Dikalova A. (2019). Tobacco smoking induces cardiovascular mitochondrial oxidative stress, promotes endothelial dysfunction, and enhances hypertension. Am. J. Physiol.-Heart Circ. Physiol..

[4] Gupta S.C., Hevia D., Patchva S., Park B., Koh W., Aggarwal B.B. (2012). Upsides and downsides of reactive oxygen species for Cancer: The roles of reactive oxygen species in tumorigenesis, prevention, and therapy. Antioxid. Redox Signal.

[5] Zorov D.B., Juhaszova M., Sollott S.J. (2014). Mitochondrial reactive oxygen species (ROS) and ROS-induced ROS release. Physiol. Rev..

[6] Tsutsui H., Kinugawa S., Matsushima S. (2011). Oxidative stress and heart failure. Am. J. Physiol.-Heart Circ. Physiol..

[7] Liguori I., Russo G., Curcio F., Bulli G., Aran L., Della-Morte D., Gargiulo G., Testa G., Cacciatore F., Bonaduce D. (2018). Oxidative stress, aging, and diseases. Clin. Interv. Aging.

[8] Sharifi-Rad M., Kumar N.V.A., Zucca P., Varoni E.M., Dini L., Panzarini E., Rajkovic J., Fokou P.V.T., Azzini E., Peluso I. (2020). Lifestyle, Oxidative Stress, and Antioxidants: Back and Forth in the Pathophysiology of Chronic Diseases. Front. Physiol..

[9] Dubois-Deruy E., Cuvelliez M., Fiedler J., Charrier H., Mulder P., Hebbar E., Pfanne A., Beseme O., Chwastyniak M., Amouyel P. (2017). MicroRNAs regulating superoxide dismutase 2 are new circulating biomarkers of heart failure. Sci. Rep..

[10] Strassburger M., Bloch W., Sulyok S., Schüller J., Keist A.F., Schmidt A., Wenk J., Peters T., Wlaschek M., Krieg T. (2005). Heterozygous deficiency of manganese superoxide dismutase results in severe lipid peroxidation and spontaneous apoptosis in murine myocardium in vivo. Free Radic. Biol. Med..

[11] Li X., Lin Y., Wang S., Zhou S., Ju J., Wang X., Chen Y., Xia M. (2020). Extracellular Superoxide Dismutase Is Associated With Left Ventricular Geometry and Heart Failure in Patients with Cardiovascular Disease. J. Am. Heart Assoc..

[12] Tehrani H.S., Moosavi-Movahedi A.A. (2018). Catalase and its mysteries. Prog. Biophys. Mol. Biol..

[13] Detienne G., De Haes W., Mergan L., Edwards S.L., Temmerman L., Van Bael S. (2018). Beyond ROS clearance: Peroxiredoxins in stress signaling and aging. Ageing Res. Rev..

[14] Kuzuya K., Ichihara S., Suzuki Y., Inoue C., Ichihara G., Kurimoto S., Oikawa S. (2018). Proteomics analysis identified peroxiredoxin 2 involved in early-phase left ventricular impairment in hamsters with cardiomyopathy. PLoS ONE.

[15] Ibarrola J., Arrieta V., Sádaba R., Martinez-Martinez E., Garcia-Peña A., Alvarez V., Fernández-Celis A., Gainza A., Santamaría E., Fernández-Irigoyen J. (2018). Galectin-3 down-regulates antioxidant peroxiredoxin-4 in human cardiac fibroblasts: A new pathway to induce cardiac damage. Clin. Sci..

[16] Cieniewski-Bernard C., Mulder P., Henry J.-P., Drobecq H., Dubois E., Pottiez G., Thuillez C., Amouyel P., Richard V., Pinet F. (2008). Proteomic Analysis of Left Ventricular Remodeling in an Experimental Model of Heart Failure. J. Proteome Res..

[17] Kalyanaraman B. (2013). Teaching the basics of redox biology to medical and graduate students: Oxidants, antioxidants and disease mechanisms. Redox Biol..

[18] Park T.J., Park J.H., Lee G.S., Lee J.Y., Shin J.H., Kim M.W., Kim Y.S., Kim J.Y., Oh K.J., Han B.S. (2019). Quantitative proteomic analyses reveal that GPX4 downregulation during myocardial infarction contributes to ferroptosis in cardiomyocytes. Cell Death Dis..

[19] Tsutsui H., Kinugawa S., Matsushima S. (2008). Mitochondrial oxidative stress and dysfunction in myocardial remodelling. Cardiovasc. Res..

[20] Sack M.N., Fyhrquist F.Y., Saijonmaa O.J., Fuster V., Kovacic J.C. (2017). Basic Biology of Oxidative Stress and the Cardiovascular System: Part 1 of a 3-Part Series. J. Am. Coll. Cardiol..

[21] Burgoyne J.R., Mongue-Din H., Eaton P., Shah A.M. (2012). Redox signaling in cardiac physiology and pathology. Circ. Res..

[22] Moris D., Spartalis M., Tzatzaki E., Spartalis E., Karachaliou G.S., Triantafyllis A.S., Karaolanis G.I., Tsilimigras D.I., Theocharis S. (2017). The role of reactive oxygen species in myocardial redox signaling and regulation. Ann. Transl. Med..

[23] Lismont C., Revenco I., Fransen M. (2019). Peroxisomal hydrogen peroxide metabolism and signaling in health and disease. Int. J. Mol. Sci..

[24] Kasai S., Shimizu S., Tatara Y., Mimura J., Itoh K. (2020). Regulation of Nrf2 by mitochondrial reactive oxygen species in physiology and pathology. Biomolecules.

[25] Loyer X., Heymes C., Samuel J.L. (2008). Constitutive nitric oxide synthases in the heart from hypertrophy to failure. Clin. Exp. Pharmacol. Physiol..

[26] Hammond J., Balligand J.L. (2012). Nitric oxide synthase and cyclic GMP signaling in cardiac myocytes: From contractility to remodeling. J. Mol. Cell. Cardiol..

[27] Lancel S., Qin F., Lennon S.L., Zhang J., Tong X., Mazzini M.J., Kang Y.J., Siwik D.A., Cohen R.A., Colucci W.S. (2010). Oxidative posttranslational modifications mediate decreased SERCA activity and myocyte dysfunction in Galphaq-overexpressing mice. Circ. Res..

[28] Steinberg S.F. (2013). Oxidative stress and sarcomeric proteins. Circ. Res..

[29] Hermida N., Michel L., Esfahani H., Dubois-Deruy E., Hammond J., Bouzin C., Markl A., Colin H., Van Steenbergen A., De Meester C. (2018). Cardiac myocyte β3-adrenergic receptors prevent myocardial fibrosis by modulating oxidant stress-dependent paracrine signaling. Eur. Heart J..

[30] Serpillon S., Floyd B.C., Gupte R.S., George S., Kozicky M., Neito V., Recchia F., Stanley W., Wolin M.S., Gupte S.A. (2009). Superoxide production by NAD(P)H oxidase and mitochondria is increased in genetically obese and hyperglycemic rat heart and aorta before the development of cardiac dysfunction. The role of glucose-6-phosphate dehydrogenase-derived NADPH. Am. J. Physiol.-Heart Circ. Physiol..

[31] Anderson E.J., Kypson A.P., Rodriguez E., Anderson C.A., Lehr E.J., Neufer P.D. (2009). Substrate-Specific Derangements in Mitochondrial Metabolism and Redox Balance in the Atrium of the Type 2 Diabetic Human Heart. J. Am. Coll. Cardiol..

[32] Fortuño A., José G.S., Moreno M.U., Beloqui O., Díez J., Zalba G. (2006). Phagocytic NADPH oxidase overactivity underlies oxidative stress in metabolic syndrome. Diabetes.

[33] Sverdlov A.L., Elezaby A., Qin F., Behring J.B., Luptak I., Calamaras T.D., Siwik D.A., Miller E.J., Liesa M., Shirihai O.S. (2016). Mitochondrial reactive oxygen species mediate cardiac structural, functional, and mitochondrial consequences of diet-induced metabolic heart disease. J. Am. Heart Assoc..

[34] Jeong E.M., Chung J., Liu H., Go Y., Gladstein S., Farzaneh-Far A., Lewandowski E.D., Dudley S.C. (2016). Role of Mitochondrial Oxidative Stress in Glucose Tolerance, Insulin Resistance, and Cardiac Diastolic Dysfunction. J. Am. Heart Assoc..

[35] Niemann B., Chen Y., Teschner M., Li L., Silber R.E., Rohrbach S. (2011). Obesity induces signs of premature cardiac aging in younger patients: The role of mitochondria. J. Am. Coll. Cardiol..

[36] Jiménez-González S., Marín-Royo G., Jurado-López R., Bartolomé M.V., Romero-Miranda A., Luaces M., Islas F., Nieto M.L., Martínez-Martínez E., Cachofeiro V. (2020). The Crosstalk between Cardiac Lipotoxicity and Mitochondrial Oxidative Stress in the Cardiac Alterations in Diet-Induced Obesity in Rats. Cells.

[37] Li J.M., Gall N.P., Grieve D.J., Chen M., Shah A.M. (2002). Activation of NADPH oxidase during progression of cardiac hypertrophy to failure. Hypertension.

[38] Dai D.F., Johnson S.C., Villarin J.J., Chin M.T., Nieves-Cintrón M., Chen T., Marcinek D.J., Dorn G.W., Kang Y.J., Prolla T.A. (2011). Mitochondrial oxidative stress mediates angiotensin II-induced cardiac hypertrophy and gαq overexpression-induced heart failure. Circ. Res..

[39] Dai D.F., Hsieh E.J., Liu Y., Chen T., Beyer R.P., Chin M.T., MacCoss M.J., Rabinovitch P.S. (2012). Mitochondrial proteome remodelling in pressure overload-induced heart failure: The role of mitochondrial oxidative stress. Cardiovasc. Res..

[40] Ide T., Tsutsui H., Hayashidani S., Kang D., Suematsu N., Nakamura K.I., Utsumi H., Hamasaki N., Takeshita A. (2001). Mitochondrial DNA damage and dysfunction associated with oxidative stress in failing hearts after myocardial infarction. Circ. Res..

[41] Merabet N., Bellien J., Glevarec E., Nicol L., Lucas D., Remy-Jouet I., Bounoure F., Dreano Y., Thuillez C., Mulder P. (2012). Soluble epoxide hydrolase inhibition improves myocardial perfusion and function in experimental heart failure. J. Mol. Cell. Cardiol..

[42] Santillo M., Colantuoni A., Mondola P., Guida B., Damiano S. (2015). NOX signaling in molecular cardiovascular mechanisms involved in the blood pressure homeostasis. Front. Physiol..

[43] Weng X., Yu L., Liang P., Li L., Dai X., Zhou B., Wu X., Xu H., Fang M., Chen Q. (2015). A crosstalk between chromatin remodeling and histone H3K4 methyltransferase complexes in endothelial cells regulates angiotensin II-induced cardiac hypertrophy. J. Mol. Cell. Cardiol..

[44] Sorescu D., Weiss D., Lassègue B., Clempus R.E., Szöcs K., Sorescu G.P., Valppu L., Quinn M.T., Lambeth J.D., Vega J.D. (2002). Superoxide production and expression of Nox family proteins in human atherosclerosis. Circulation.

[45] Erickson J.R., Mei-ling A.J., Guan X., Kutschke W., Yang J., Oddis C.V., Bartlett R.K., Lowe J.S., O’Donnell S.E., Aykin-Burns N. (2008). A Dynamic Pathway for Calcium-Independent Activation of CaMKII by Methionine Oxidation. Cell.

[46] Scioli M.G., Storti G., D’Amico F., Rodríguez Guzmán R., Centofanti F., Doldo E., Miranda E.M.C., Orlandi A. (2020). Oxidative Stress and New Pathogenetic Mechanisms in Endothelial Dysfunction: Potential Diagnostic Biomarkers and Therapeutic Targets. J. Clin. Med..

[47] Umar S., Van Der Laarse A. (2010). Nitric oxide and nitric oxide synthase isoforms in the normal, hypertrophic, and failing heart. Mol. Cell. Biochem..

[48] Cai H., Harrison D.G. (2000). Endothelial dysfunction in cardiovascular diseases: The role of oxidant stress. Circ. Res..

[49] Ma L., Wang K., Shang J., Cao C., Zhen P., Liu X., Wang W., Zhang H., Du Y., Liu H. (2014). Anti-peroxynitrite treatment ameliorated vasorelaxation of resistance arteries in aging rats: Involvement with NO-sGC-cGKs pathway. PLoS ONE.

[50] Mollnau H., Oelze M., August M., Wendt M., Daiber A., Schulz E., Baldus S., Kleschyov A.L., Materne A., Wenzel P. (2005). Mechanisms of increased vascular superoxide production in an experimental model of idiopathic dilated cardiomyopathy. Arterioscler. Thromb. Vasc. Biol..

[51] Scialò F., Fernández-Ayala D.J., Sanz A. (2017). Role of mitochondrial reverse electron transport in ROS signaling: Potential roles in health and disease. Front. Physiol..

[52] Mazat J.P., Devin A., Ransac S. (2020). Modelling mitochondrial ROS production by the respiratory chain. Cell. Mol. Life Sci..

[53] Bhatti J.S., Bhatti G.K., Reddy P.H. (2017). Mitochondrial dysfunction and oxidative stress in metabolic disorders—A step towards mitochondria based therapeutic strategies. Biochim. Biophys. Acta-Mol. Basis Dis..

[54] Niemann B., Rohrbach S., Miller M.R., Newby D.E., Fuster V., Kovacic J.C. (2017). Oxidative Stress and Cardiovascular Risk: Obesity, Diabetes, Smoking, and Pollution: Part 3 of a 3-Part Series. J. Am. Coll. Cardiol..

[55] Bugger H., Pfeil K. (2020). Mitochondrial ROS in myocardial ischemia reperfusion and remodeling. Biochim. Biophys. Acta-Mol. Basis Dis..

[56] Rababa’h A.M., Guillory A.N., Mustafa R., Hijjawi T. (2018). Oxidative Stress and Cardiac Remodeling: An Updated Edge. Curr. Cardiol. Rev..

[57] Gori T., Münzel T. (2011). Oxidative stress and endothelial dysfunction: Therapeutic implications. Ann. Med..

[58] Stamler J.S., Osborne J.A., Jaraki O., Rabbani L.E., Mullins M., Singel D., Loscalzo J. (1993). Adverse vascular effects of homocysteine are modulated by endothelium-derived relaxing factor and related oxides of nitrogen. J. Clin. Investig..

[59] McCully K.S. (1992). Homocystinuria, Arteriosclerosis, Methylmalonic Aciduria, and Methyltransferase Deficiency: A Key Case Revisited. Nutr. Rev..

[60] Cianciolo G., De Pascalis A., Di Lullo L., Ronco C., Zannini C., La Manna G. (2017). Folic acid and homocysteine in chronic kidney disease and cardiovascular disease progression: Which comes first?. CardioRenal Med..

[61] Stroes E.S.G., Van Faassen E.E., Yo M., Martasek P., Boer P., Govers R., Rabelink T.J. (2000). Folic acid reverts dysfunction of endothelial nitric oxide synthase. Circ. Res..

[62] Hagar H.H. (2002). Folic acid and vitamin B12 supplementation attenuates isoprenaline-induced myocardial infarction in experimental hyperhomocysteinemic rats. Pharmacol. Res..

[63] Farhangi M.A., Nameni G., Hajiluian G., Mesgari-Abbasi M. (2017). Cardiac tissue oxidative stress and inflammation after vitamin D administrations in high fat- diet induced obese rats. BMC Cardiovasc. Disord..

[64] Leme Goto P., Cinato M., Merachli F., Vons B., Jimenez T., Marsal D., Todua N., Loi H., Santin Y., Cassel S. (2020). In vitro and in vivo cardioprotective and metabolic efficacy of vitamin E TPGS/Apelin. J. Mol. Cell. Cardiol..

[65] Wallert M., Ziegler M., Wang X., Maluenda A., Xu X., Yap M.L., Witt R., Giles C., Kluge S., Hortmann M. (2019). α-Tocopherol preserves cardiac function by reducing oxidative stress and inflammation in ischemia/reperfusion injury. Redox Biol..

[66] Banez M.J., Geluz M.I., Chandra A., Hamdan T., Biswas O.S., Bryan N.S., Von Schwarz E.R. (2020). A systemic review on the antioxidant and anti-inflammatory effects of resveratrol, curcumin, and dietary nitric oxide supplementation on human cardiovascular health. Nutr. Res..

[67] Agouni A., Lagrue-Lak-Hal A.H., Mostefai H.A., Tesse A., Mulder P., Rouet P., Desmoulin F., Heymes C., Martínez M.C., Andriantsitohaina R. (2009). Red wine polyphenols prevent metabolic and cardiovascular alterations associated with obesity in Zucker fatty rats (Fa/Fa). PLoS ONE.

[68] Farkhondeh T., Folgado S.L., Pourbagher-Shahri A.M., Ashrafizadeh M., Samarghandian S. (2020). The therapeutic effect of resveratrol: Focusing on the Nrf2 signaling pathway. Biomed. Pharmacother..

[69] Dyck G.J.B., Raj P., Zieroth S., Dyck J.R.B., Ezekowitz J.A. (2019). The effects of resveratrol in patients with cardiovascular disease and heart failure: A narrative review. Int. J. Mol. Sci..

[70] Huang J.P., Hsu S.C., Li D.E., Chen K.H., Kuo C.Y., Hung L.M. (2018). Resveratrol mitigates high-fat diet-induced vascular dysfunction by activating the Akt/eNOS/NO and Sirt1/ER pathway. J. Cardiovasc. Pharmacol..

[71] Chan A.Y.M., Dolinsky V.W., Soltys C.-L.M., Viollet B., Baksh S., Light P.E., Dyck J.R.B. (2008). Resveratrol inhibits cardiac hypertrophy via AMP-activated protein kinase and Akt. J. Biol. Chem..

[72] Sung M.M., Das S.K., Levasseur J., Byrne N.J., Fung D., Kim T.T., Masson G., Boisvenue J., Soltys C.L., Oudit G.Y. (2015). Resveratrol treatment of mice with pressure-overloadinduced heart failure improves diastolic function and cardiac energy metabolism. Circ. Heart Fail..

[73] Tanno M., Kuno A., Yano T., Miura T., Hisahara S., Ishikawa S., Shimamoto K., Horio Y. (2010). Induction of manganese superoxide dismutase by nuclear translocation and activation of SIRT1 promotes cell survival in chronic heart failure. J. Biol. Chem..

[74] Sabbah H.N. (2016). Targeting mitochondrial dysfunction in the treatment of heart failure. Expert Rev. Cardiovasc. Ther..

[75] Senoner T., Dichtl W. (2019). Oxidative stress in cardiovascular diseases: Still a therapeutic target?. Nutrients.

[76] Dikalova A.E., Bikineyeva A.T., Budzyn K., Nazarewicz R.R., McCann L., Lewis W., Harrison D.G., Dikalov S.I. (2010). Therapeutic targeting of mitochondrial superoxide in hypertension. Circ. Res..

[77] Ni R., Cao T., Xiong S., Ma J., Fan G.C., Lacefield J.C., Lu Y., Tissier S.L., Peng T. (2016). Therapeutic inhibition of mitochondrial reactive oxygen species with mito-TEMPO reduces diabetic cardiomyopathy. Free Radic. Biol. Med..

[78] Kelso G.F., Porteous C.M., Coulter C.V., Hughes G., Porteous W.K., Ledgerwood E.C., Smith R.A.J., Murphy M.P. (2001). Selective targeting of a redox-active ubiquinone to mitochondria within cells: Antioxidant and antiapoptotic properties. J. Biol. Chem..

[79] Kim S., Song J., Ernst P., Latimer M.N., Ha C.M., Goh K.Y., Ma W., Rajasekaran N.S., Zhang J., Liu X. (2020). MitoQ regulates redox-related noncoding RNAs to preserve mitochondrial network integrity in pressure-overload heart failure. Am. J. Physiol. Heart Circ. Physiol..

[80] Rao V.A., Klein S.R., Bonar S.J., Zielonka J., Mizuno N., Dickey J.S., Keller P.W., Joseph J., Kalyanaraman B., Shacter E. (2010). The antioxidant transcription factor Nrf2 negatively regulates autophagy and growth arrest induced by the anticancer redox agent mitoquinone. J. Biol. Chem..

[81] Doughan A.K., Dikalov S.I. (2007). Mitochondrial redox cycling of mitoquinone leads to superoxide production and cellular apoptosis. Antioxid. Redox Signal.

[82] Pokrzywinski K.L., Biel T.G., Kryndushkin D., Rao V.A. (2016). Therapeutic targeting of the mitochondria initiates excessive superoxide production and mitochondrial depolarization causing decreased mtDNA integrity. PLoS ONE.

[83] Lee I.M., Cook N.R., Gaziano J.M., Gordon D., Ridker P.M., Manson J.A.E., Hennekens C.H., Buring J.E. (2005). Vitamin E in the primary prevention of cardiovascular disease and cancer. The women’s health study: A randomized controlled trial. J. Am. Med. Assoc..

[84] Toole J.F., Malinow M.R., Chambless L.E., Spence J.D., Pettigrew L.C., Howard V.J., Sides E.G., Wang C.H., Stampfer M. (2004). Lowering Homocysteine in Patients with Ischemic Stroke to Prevent Recurrent Stroke, Myocardial Infarction, and Death: The Vitamin Intervention for Stroke Prevention (VISP) Randomized Controlled Trial. J. Am. Med. Assoc..

[85] Lonn E. (2005). Effects of long-term vitamin E supplementation on cardiovascular events and cancer: A randomized controlled trial. J. Am. Med. Assoc..

[86] Bønaa K.H., Njølstad I., Ueland P.M., Schirmer H., Tverdal A., Steigen T., Wang H., Nordrehaug J.E., Arnesen E., Rasmussen K. (2006). Homocysteine lowering and cardiovascular events after acute myocardial infarction. N. Engl. J. Med..

[87] Münzel T., Camici G.G., Maack C., Bonetti N.R., Fuster V., Kovacic J.C. (2017). Impact of Oxidative Stress on the Heart and Vasculature: Part 2 of a 3-Part Series. J. Am. Coll. Cardiol..

[88] Mankowski R.T., You L., Buford T.W., Leeuwenburgh C., Manini T.M., Schneider S., Qiu P., Anton S.D. (2020). Higher dose of resveratrol elevated cardiovascular disease risk biomarker levels in overweight older adults–A pilot study. Exp. Gerontol..

[89] Tomé-Carneiro J., Gonzálvez M., Larrosa M., Yáñez-Gascón M.J., García-Almagro F.J., Ruiz-Ros J.A., García-Conesa M.T., Tomás-Barberán F.A., Espín J.C. (2012). One-year consumption of a grape nutraceutical containing resveratrol improves the inflammatory and fibrinolytic status of patients in primary prevention of cardiovascular disease. Am. J. Cardiol..

[90] Macedo R.C.S., Vieira A., Marin D.P., Otton R. (2015). Effects of chronic resveratrol supplementation in military firefighters undergo a physical fitness test-A placebo-controlled, double blind study. Chem. Biol. Interact..

[91] Timmers S., Konings E., Bilet L., Houtkooper R.H., Van De Weijer T., Goossens G.H., Hoeks J., Van Der Krieken S., Ryu D., Kersten S. (2011). Calorie restriction-like effects of 30 days of resveratrol supplementation on energy metabolism and metabolic profile in obese humans. Cell Metab..

[92] Fujitaka K., Otani H., Jo F., Jo H., Nomura E., Iwasaki M., Nishikawa M., Iwasaka T., Das D.K. (2011). Modified resveratrol Longevinex improves endothelial function in adults with metabolic syndrome receiving standard treatment. Nutr. Res..

[93] Imamura H., Yamaguchi T., Nagayama D., Saiki A., Shirai K., Tatsuno I. (2017). Resveratrol ameliorates arterial stiffness assessed by cardio-ankle vascular index in patients with type 2 diabetes mellitus. Int. Heart J..

[94] Van Der Made S.M., Plat J., Mensink R.P. (2015). Resveratrol does not influence metabolic risk markers related to cardiovascular health in overweight and slightly obese subjects: A randomized, placebo-controlled crossover trial. PLoS ONE.

[95] Olesen J., Gliemann L., Biensø R., Schmidt J., Hellsten Y., Pilegaard H. (2014). Exercise training, but not resveratrol, improves metabolic and inflammatory status in skeletal muscle of aged men. J. Physiol..

[96] Agarwal B., Campen M.J., Channell M.M., Wherry S.J., Varamini B., Davis J.G., Baur J.A., Smoliga J.M. (2013). Resveratrol for primary prevention of atherosclerosis: Clinical trial evidence for improved gene expression in vascular endothelium. Int. J. Cardiol..

